# CD4^+^ T Cell-Derived IL-2 Signals during Early Priming Advances Primary CD8^+^ T Cell Responses

**DOI:** 10.1371/journal.pone.0007766

**Published:** 2009-11-10

**Authors:** Yo-Ping Lai, Chia-Ching Lin, Wan-Jung Liao, Chih-Yung Tang, Shu-Ching Chen

**Affiliations:** 1 Department of Internal Medicine, National Taiwan University Hospital, Taipei, Taiwan; 2 Department of Medical Research, National Taiwan University Hospital, Taipei, Taiwan; 3 Graduate Institute of Physiology, College of Medicine, National Taiwan University, Taipei, Taiwan; University of Toronto, Canada

## Abstract

Stimulating naïve CD8^+^ T cells with specific antigens and costimulatory signals is insufficient to induce optimal clonal expansion and effector functions. In this study, we show that the activation and differentiation of CD8^+^ T cells require IL-2 provided by activated CD4^+^ T cells at the initial priming stage within 0–2.5 hours after stimulation. This critical IL-2 signal from CD4^+^ cells is mediated through the IL-2Rβγ of CD8^+^ cells, which is independent of IL-2Rα. The activation of IL-2 signaling advances the restriction point of the cell cycle, and thereby expedites the entry of antigen-stimulated CD8^+^ T-cell into the S phase. Besides promoting cell proliferation, IL-2 stimulation increases the amount of IFNγ and granzyme B produced by CD8^+^ T cells. Furthermore, IL-2 at priming enhances the ability of P14 effector cells generated by antigen activation to eradicate B16.gp33 tumors *in vivo*. Therefore, our studies demonstrate that a full CD8^+^ T-cell response is elicited by a critical temporal function of IL-2 released from CD4^+^ T cells, providing mechanistic insights into the regulation of CD8^+^ T cell activation and differentiation.

## Introduction

Upon encountering their antigens, naïve T cells are activated and driven to clonal expansion and differentiation into armed effector cells, the cytotoxic T cells (CTL). The CTL capable of producing cytokines and cytotoxic mediators are armed to kill transformed, virus-infected, or allogeneic cells [1], [2]. According to the two-signal hypothesis, the induction of an optimal CD4^+^ T-cell immune response requires both antigen-specific and co-stimulatory signals [3], [4], [5]. In contrast to the CD4^+^ T-cell activation, stimulation by specific antigens and costimulatory signal fails to drive naïve CD8^+^ T cells to clonal expansion and differentiation [6]. Thus, CD8^+^ T cells require additional signals for full activation and further differentiation into effector cells.

The role of CD4^+^ T cells in CD8^+^ T-cell activation has not been fully understood. It is reported that CD8^+^-mediated allograft rejection requires CD4+ T-cell help [7] but primary CD8^+^ CTL responses to infectious agents can be readily detectable in hosts that lack CD4^+^ T cells [8], [9], [10]. However, these CD8^+^ T-cell primed in the absence of CD4^+^ T cells are not capable of mounting an effective secondary response [11], [12], indicating that CD4^+^ T cells are required for an effective CD8^+^ T-cell activation and differentiation.

A number of models were put forth to explain how of CD4^+^ T cells help for CD8^+^ T-cell responses. Studies have shown that CD4^+^ T-cell help is important for the programming of CD8^+^ T-cell differentiation [13], [14], [15], [16]. Also, by secreting interleukin-2 (IL-2), CD4^+^ T cells act at later time points to maintain CD8^+^ T-cell growth [17], [18], [19]. Recently, the “three-cell model” depicts that CD4^+^ T cells and CD8^+^ T cells are brought together on the same antigen-presenting cell (APC) for effective delivery of CD4^+^ T-cell help [20], [21]. CD4^+^ T cells condition APCs to increase their ability to stimulate CD8^+^ T cells [20], [22], [23], [24], [25], which may involve a direct CD40-CD40L interaction between CD4^+^ and CD8^+^ cells [11]. However, several important questions remain unanswered. First, the CD4^+^ dependence of primary CTL responses cannot be concluded firmly due to the possibility that the primary CTL responses to non-infectious antigens are too weak to be measured by direct ex vivo killing assays. Second, the temporal action of CD4^+^ T cells for the activation and differentiation of CD8^+^ cells remains undefined. Third, there is controversy that some studies have found no critical role for CD40 molecules in primary or memory CD8^+^ T cell responses [14], [26]. Fourth, it is unknown whether the CD4^+^-dependent activation of CD8^+^ T cells requires cell-cell contact or secreting mediators.

To address these issues, we hypothesized that CD4^+^ T cells may directly provide help for a CD8^+^ T cell response. We adopted an *in vitro* approach to dissect the cellular and molecular requirements for CD8^+^ T-cell activation and differentiation. Naïve CD62L^hi^CD44^lo^ CD8^+^ T cells were sorted and stimulated by anti-CD3 and anti-CD28 antibodies. This method allowed us to study the critical time point when CD4^+^ T cells help for eliciting a CD8^+^ T response and to investigate which soluble mediator(s) is required for naïve CD8^+^ T-cell activation during priming. Here, we demonstrate that during the early priming stage, the IL-2 produced by activated CD4^+^ T cells promotes the CD8^+^ T-cell activation and differentiation. Moreover, the IL-2 signal in priming stage facilitates the proliferation of CD8^+^ T cells by advancing the restriction point of the cell cycle. We also showed that IL-2 at priming enhances the ability of CD8+ T cells to eradicate tumor by producing greater quantities of interferon-γ and granzyme B. These findings takes us one step closer to the understanding of how CD4^+^ T cells help CD8^+^ T-cell activation and define the temporal function of IL-2 in regulation of a CD8^+^ T cell response.

## Results

### IL-2 Produced by Activated CD4^+^ T Cells Helps CD8^+^ T-Cell Activation

**Figure 1:**
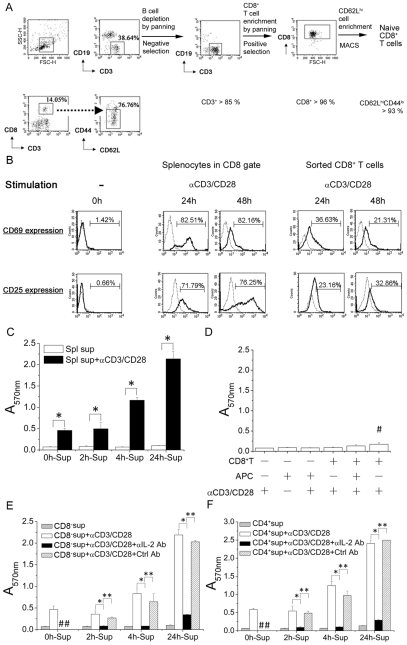
Activated CD4^+^ T cells secreted IL-2 to help primary CD8^+^ T-cell activation. (A) Naïve CD8^+^ T cells (CD62L^hi^CD44^lo^) from B6 mice spleen with purity above 93% were obtained through B cell depletion, CD8^+^ T cells enrichment by panning and further enriched by MACS sorting with anti-CD62L MACS beads. (B) Expression of activation markers, CD69 and CD25. 3×10^6^/mL of naïve CD8^+^ T cells (left) were stimulated by anti-CD3/CD28 antibodies for 0 hour and splenocytes (middle) or naïve CD8^+^ T cells (right) in 24-well culture plate were stimulated by anti-CD3/CD28 antibodies for indicated time, 24 and 48 hours and harvested, followed by staining with FITC anti-CD25, PE anti-CD69 and PE-Cy5 anti-CD8 antibodies, and analysis by flow cytometry. Dotted line: Isotype control. (C) Naïve CD8^+^ T cells (1×10^5^/well) in a 96-well culture plate were stimulated in the presence or absence of mitomycin C-treated splenocytes (APC) for 96 hours, followed by MTT assay (numbers represent OD at 570 nm). The effect of CD4-depleted splenocytes was also checked (#). (D) Culture supernatants of anti-CD3/CD28 stimulated splenocytes for the indicated time, 0, 2, 4 and 24 hours (0 h-, 2 h-, 4 h- and 24 h-Sup) were collected. Naïve CD8^+^ T cells (3×10^5^/well) in the 96-well culture plate were then incubated with the culture supernatant with (▪) or without (□) stimulation by anti-CD3/CD28 antibodies. Following 96 hours of stimulation, the cell viability was measured by MTT assay. (E and F) Naïve CD8^+^ T cells (3×10^5^/well) in the 96-well culture plate were stimulated in the presence of culture supernatants of 0 h-, 2 h-, 4 h- and 24 h-stimulated CD8^−^ splenocytes (E) or 0 h-, 2 h-, 4 h- and 24 h-stimulated CD4^+^ T cells (F) for 96 hours. Anti-mouse IL-2 antibody (2.5 µg/mL) was used to neutralize murine IL-2 in culture supernatant. The cell viability was measured by MTT assay. The data represent three independent experiments. #, The groups in which the cells received anti-IL-2 or isotype control antibody were not included. *, **, significantly statistical difference by student *t*-test, *p<0.05*. APC, antigen-presenting cells. CD8^−^, CD8^+^-depleted splenocytes. Spl, splenocytes. Sup, culture supernatant. Ab, antibody. Ctrl, isotype control of rat anti-murine IL-2 antibody, rat IgG_2a_.

To study the requirement of naïve CD8^+^ T cell activation, we sorted CD62L^hi^CD44^lo^ CD8^+^ T cells (Figure 1A) and compared them to splenocytes. Within 24 and 48 hours after stimulation, we found that the CD69 and CD25 activation molecules were significantly up-regulated in the population of splenocytes within CD8^+^ gate (Figure 1B, middle panels). However, the purified naïve CD8^+^ T cells showed poor activation under the same condition (Figure 1B, right panels). These results suggest that factors other than CD8^+^ T-cell in the mixed splenocyte population help CD8^+^ T-cell activation. To assess whether soluble mediator(s) secreted by splenocytes plays a helper role. Supernatants collected from cultures harvested at 0, 2, 4 and 24 hours after stimulation (0h-Sup, 2h-Sup, 4h-Sup and 24h-Sup) were added to purified naïve CD8^+^ T cell cultures. MTT assay showed that there were significantly more viable CD8^+^ T cells in the wells containing supernatants collected from splenocyte cultures with longer stimulation time. These results suggest that soluble factor(s) in the supernatant helps CD8^+^ T cells to respond to stimulation expand and survive (Figure 1C). The next question was whether cellular factor(s), such as antigen-presenting cells (APC), plays a helper role. We used mitomycin C-treated splenocytes as a source of APC [27], [28], [29]. To avoid the soluble mediator(s) from CD4^+^ cell and consider the effect of CD4^−^ splenocytes, we stimulated the naïve CD8^+^ T cells in the presence of CD4-depleted splenocytes. The results show that naïve CD8^+^ T cells stimulated by anti-CD3/CD28 antibodies in the presence of mitomycin C-treated splenocytes or CD4^+^-depleted splenocytes were only minimally activated (Figure 1D). Thus, helper role of APC and CD4-depleted splenocytes is limited.

To determine the cellular source of the soluble mediator(s), we collected supernatants from purified CD4^+^ T cell and from CD8^−^ splenocytes cultures at 0, 2, 4, or 24 hours after anti-CD3/CD28 stimulation. Naïve CD8^+^ T cells cultured in the different supernatants were stimulated by anti-CD3/CD28 antibodies. As demonstrated in Figure 1E and 1F, the effects of supernatants collected from CD8^−^ splenocytes and purified CD4^+^ T cells were comparable in helping CD8^+^ T cell expansion and survival, indicating that CD4^+^ T cells may be the source of soluble mediator(s). ELISA results showed that supernatants from cultures of total splenocytes, CD8^−^ splenocytes, and purified CD4^+^ T cells stimulated with anti-CD3/CD28 antibodies contained high IL-2 levels and the levels of IL-2 were comparable (Table 1). Moreover, compared to treatment with isotype control rat IgG_2a_, neutralizing IL-2 by neutralizing rat anti-murine IL-2 antibody in the culture supernatants abolished the help provided in the culture supernatants to CD8^+^ T cell activation (Figures 1E and 1F). These results together suggest that IL-2 as produced by CD4 T cells is the soluble factor that helps CD8^+^ T cell activation.

**Table 1:** IL-2 concentration in culture supernatants of stimulated splenocytes, CD8-depleted splenocytes and purified CD4^+^ T cells.

Time from stimulation (h)	IL-2 concentration (pg/mL)		
	*splenocytes*	*CD8^−^ splenocytes*	*CD4* ^+^ *cells*
0	N.D.*	N.D.*	N.D.*
2	16.5	14.1	26.7
4	222.9	140.3	435.3
6	972.3	873.8	662.2
24	8167.9	7553.3	11841.2

*N.D., not detectable.

### IL-2 Is Critical to CD8^+^ T Cell Activation at Early Time Points during Priming

**Figure 2:**
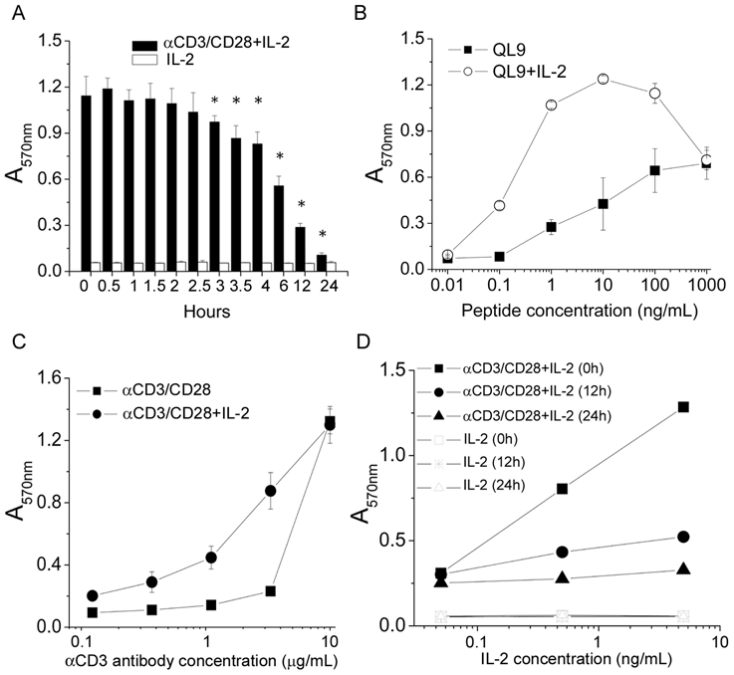
The time and amount of IL-2 required for promoting CD8^+^ T-cell activation. (A) Recombinant human IL-2 (rhIL-2, 5 ng/mL) was administrated at indicated time following CD8^+^ T-cell stimulation by anti-CD3/CD28 antibodies. Following 96 hours, cell viability was measured by MTT assay at absorbance of 570 nm (▪). The cells were incubated with rhIL-2 alone as control (□). (B) In the presence (○) or absence (▪) of rhIL-2 in priming stage, naive CD8^+^ T cells from 2C TCR transgenic mice were stimulated with specific peptide, QL9 in titrations for 96 hours, followed by MTT assay. (C) In the presence (•) or absence (▪) of rhIL-2 in priming stage, naïve B6 CD8^+^ T cells were stimulated by anti-CD3 in titrated concentrations for 96 hours, followed by MTT assay. (D) rhIL-2 in titrated concentrations, 5, 0.5 or 0.05 ng/mL was administered to the CD8^+^ T cells at 0 (▪), 12 (•) and 24 (▴) hours after stimulation by anti-CD3/CD28 antibodies. The cells were incubated with rhIL-2 alone as controls. Following 96 hours, cell viability was measured by MTT assay. The data represent three independent experiments. *, significantly statistical difference by student *t*-test, *p<0.05*.

Human IL-2 binds murine IL-2 receptor to transduce IL-2 signal in murine T cells [30]. To study the effect of IL-2 in CD8^+^ T-cell activation, we added human recombinant IL-2 (rhIL-2) to naïve murine CD8^+^ T cells at different time points after anti-CD3/CD28 stimulation. While IL-2 alone did not have any effect on CD8^+^ T cells, IL-2 added between 0 to 2.5 hours after anti-CD3/CD28 stimulation (early priming stage) resulted in greater numbers of viable CD8^+^ T cells than that added at later than 3 hours (later priming stage) (*p<0.05*) after stimulation (Figure 2A). IL-2 added to 2C TCR transgenic CD8^+^ T cell culture stimulated with specific peptide QL9 at early priming stage also enhanced antigen-specific CD8^+^ T-cell expansion (Figure 2B). These results together showed that IL-2 plays an important role during early priming in CD8^+^ T cell activation through either nonspecific anti-CD3/CD28 or specific antigenic stimulation. Moreover, the effect of IL-2 on CD8^+^ T cell expansion was obvious at low concentrations of nonspecific and specific antigenic stimulants (Figure 2B and 2C) and that it had a dose-dependent effect (Figure 2D). It is of note that lower concentrations of IL-2 added at earlier time points had a better effect than higher concentrations at later time points. These data showed that IL-2 exposure during priming through TCR and costimulation pathways transduces signal(s) critical to CD8^+^ T cell activation/expansion.

### The Action of IL-2 on CD8^+^ T Cells Is through IL-2R

It is reported that IL-2 has an anti-apoptotic effect on activated T cells [31], [32]. To clarify whether the effect of IL-2 we observed on CD8^+^ T cell activation was a result of anti-apoptotic effect, we compared the cell death rates between stimulated CD8^+^ T cells cultured in the presence and absence of rhIL-2. Flow cytometric analysis showed that there was no significant difference in the percentage of cell death between these two groups at 3.5, 12 and 24 hours after stimulation (Table 2). The cells kept at 4°C were used as negative control. Since rhIL-2 added between 0 to 2.5 hours after anti-CD3/CD28 stimulation (early priming stage) resulted in greater numbers of viable CD8^+^ T cells than that added at later than 3 hours (later priming stage) (*p<0.05*) after stimulation (Figure 2A), the effect of rhIL-2 at early priming of CD8^+^ T cell activation may not be through an anti-apoptotic mechanism.

**Table 2:** Rate of Annexin V positive in CD8^+^ T cells under various stimulation.

	Positive Annexin V (%)			
Time from stimulation (h)	*αCD3/CD28*	*αCD3/CD28*+*IL-2*	*IL-2*	*Cells kept at 4°C*
3.5	41	37	40	15
12	54	54	51	22
24	62	61	67	30

**Figure 3:**
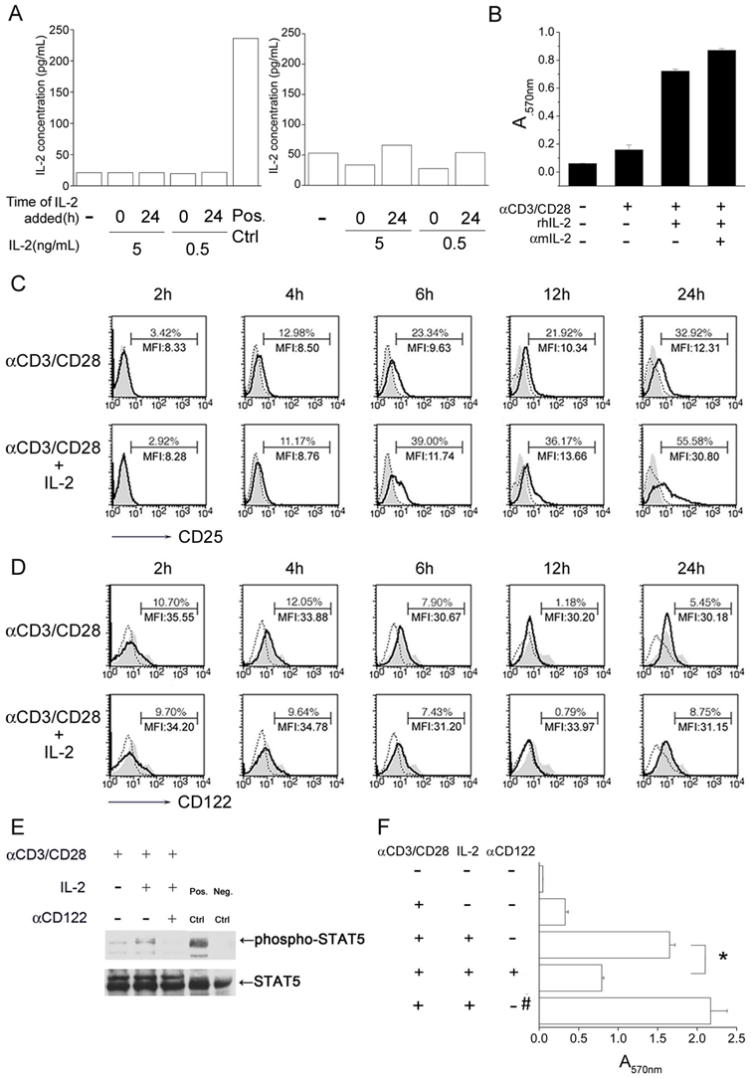
IL-2 signals to naïve CD8^+^ T cells through IL2R. (A) Naïve CD8^+^ T cells (3×10^6^/mL) were stimulated by anti-CD3/CD28 antibodies in the presence or absence of rhIL-2 at priming (0 hour) or 24 hours after priming. At 24 (left) and 48 (right) hours following stimulation, murine IL-2 level in culture supernatant was checked by ELISA. The culture supernatant of 24 hours-stimulated splenocytes was used as positive control. (B) Neutralizing anti-mouse IL-2 (2.5 µg/mL) was added simultaneously with rhIL-2 (5 ng/mL) upon the naïve CD8^+^ T cell stimulation, followed by MTT assay 72 hours after stimulation. (C and D) At indicated time, 2, 4, 6, 12 and 24 hours following stimulation by anti-CD3/CD28 antibodies, CD8^+^ T cells were harvested and stained by FITC anti-mouse CD25 (C) or FITC anti-mouse CD122 (D) antibody and analyzed by flow cytometry (Solid line). Dotted line: Isotype control. Gray filled: Naïve CD8^+^ T cells. The percent of cells which have CD25 or CD122 expression in the population and mean fluorescence intensity (MFI) were shown. (E) Following stimulation for 120 min, phospho-stat5 was analyzed by Western blotting. rhIL-2 (5 ng/mL) and blocking anti-CD122 (20 µg/mL) were added. Positive control: Hela cells treated by IFNα. Negative control: Hela cells without treatment. (F) Blocking anti-CD122 antibody and isotype control rat IgG_2b_ (#) was administered simultaneously with rhIL-2 to naïve CD8^+^ T cells upon stimulation by anti-CD3/CD28 antibodies, followed by MTT assay 96 hours after stimulation. Similar results were obtained in three independent experiments. The data represent three independent experiments. *, significantly statistical difference by student *t*-test, *p<0.05*. Ctrl, control.

To exclude the possibility that the effect of IL-2 signaling on CD8^+^ T-cells is triggering greater production of IL-2, we added rhIL-2 to naïve CD8^+^ T cell culture and determined the concentrations of murine IL-2 in the culture supernatants at 24 (Figure 3A, left panel) and 48 hours (Figure 3A, right panel) after stimulation. Figures 3A shows that exogenous rhIL-2 did not induce stimulated CD8^+^ T cells to produce more murine IL-2 whenever addition at 0 hour or 24 hours after stimulation. We have found that IL-2 signal at early priming triggers CD8^+^ T-cells expansion (Figure 2A). Thus, to further check whether the cells expansion is induced by IL-2 production from the anit-CD3/CD28- and rhIL-2- activated CD8^+^ T-cells, we added anti-murine IL-2 antibody to neutralize endogenous murine IL-2. The results showed neutralization of murine IL-2 did not ablate the effect of rhIL-2 on CD8^+^ T-cell viability at 72 hours after stimulation (Figures 3B), demonstrating that the function of exogenous IL-2 is not to induce more murine IL-2 production.

It has been reported that upon stimulation, the α-chain of IL-2 receptor (IL-2Rα, CD25) is induced and associates with IL-2Rβ (CD122) and IL-2Rγ (CD132) to form a high-affinity receptor [33]. To determine whether IL-2 at priming induces the up-regulation of IL-2 receptor subunits, we harvested CD8^+^ T cells at different time points after stimulation by anti-CD3/CD28 antibodies and analyzed CD25 expression by flow cytometry. We found that surface CD25 expression was induced at as early as 4 hours and IL-2 further up-regulated its expression at 6, 12 and 24 hours after stimulation (Figure 3C). Interestingly, IL-2 receptor β-chain expression was not altered by IL-2 (Figure 3D). Since the action of IL-2 on CD8^+^ T cell expansion was most effective during the first 2.5 hours after stimulation (Figure 2A), CD25 up-regulation may not be responsible for the IL-2 signal in early priming stage.

Naïve CD8^+^ T cells bear intermediate-affinity receptor (IL-2Rβγ) for IL-2 [34]. IL-2 is known to regulate T cell function by activating transcription factor STAT5 [35]. To investigate whether IL-2 transduces signals through intermediate-affinity receptor IL-2Rβγ during CD8^+^ T cell priming, we stimulated naïve CD8^+^ T cells and studied the phosphorylation of STAT5 after stimulation. Figure 3E demonstrates that exposure to IL-2 induced CD8^+^ T cell STAT5 phosphorylation and the effect of IL-2 was diminished when CD122 was blocked. Concomitantly, blocking CD122 also impeded CD8^+^ T cell expansion (Figure 3F). Taken together, these results indicate that at the priming stage of CD8^+^ T cell activation, IL-2 delivers a functional signal through IL-2Rβγ that is independent of IL-2Rα.

### IL-2 Signaling Advances CD8^+^ T Cells through S Phase and Accelerates the Cells Entering Cell Cycle

**Figure 4:**
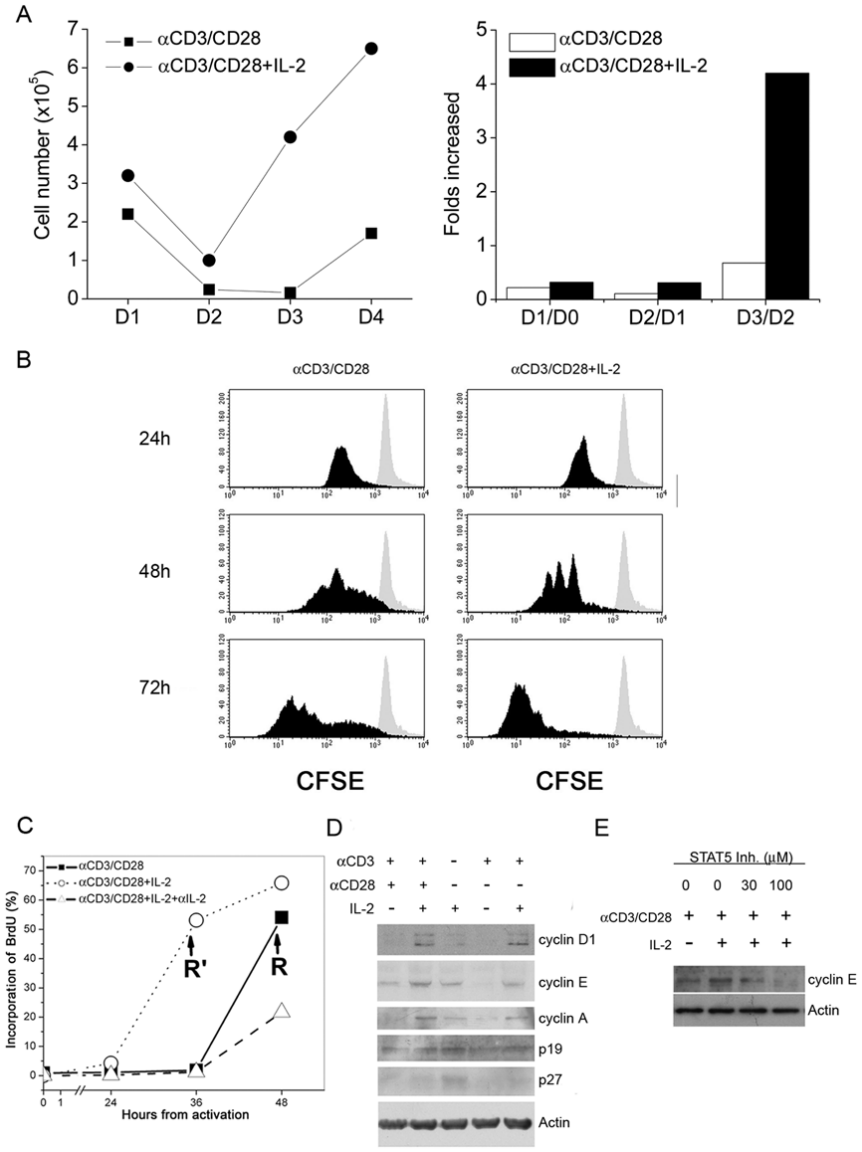
Regulation of cell cycle by IL-2 signals at priming. (A) In the presence (•) or absence (▪) of rhIL-2 (5 ng/mL) at priming, the anti-CD3/CD28-stimulated CD8^+^ T cells were counted day by day (left). Folds increase was determined day by day (right). (B) In the presence or absence of rhIL-2 at priming, CFSE-labeled naïve CD8^+^ T cells were stimulated by anti-CD3/CD28 antibodies for indicated time, 24, 48 and 72 hours, and cell divisions were analyzed by flow cytometry (black filled). Gray filled: CFSE-labeled naïve CD8^+^ T cells. (C) Naïve CD8^+^ T cells were stimulated by anti-CD3/CD28 antibodies for the indicated time, 0, 24, 36 and 48 hours, and analyzed by flow cytometry for the BrdU incorporation to determine the percentage of cell entering S phase (Solid line). Cells stimulated by anti-CD3/CD28 antibodies also with rmIL-2 administration at priming were shown as a dotted line. Dashed line shows cells additionally treated by rmIL-2 and anti-mouse IL-2 at priming. R and R' stands for restriction point. (D) Total cell extracts of 1.5×10^6^ CD8^+^ T cells at 18 hours after stimulation were collected for further Immunoblotting with antibodies against cyclins A, D1 and E, p19, p27 and actin, respectively. IL-2 (5 ng/mL) was added at priming. The data represent three independent experiments. (D  =  day)

We hypothesized that IL-2 may help CD8^+^ T cell activation through enhancing the signal that drives proliferation. To test this hypothesis, we monitored cell numbers on a daily basis for four consecutive days. Figure 4A shows that CD8^+^ T cell number was 4-fold higher in the group treated with IL-2 than that without IL-2 at 3 days after anti-CD3/CD28-stimulation. The results of CFSE analysis showed that CD8^+^ T cells cultured in the presence of exogenous IL-2 divided more vigorously than those in the absence of IL-2 (Figure 4B). To study whether greater number of cell division is due to higher number of cells entering cell cycle, we used BrdU incorporation assay to assess the number of cells entering the S phase, the restriction point, of cell cycle. Results in Figure 4C show that it took 48 hours after stimulation for 50% of CD8^+^ T cells cultured in the absence of IL-2 to enter S phase and addition of IL-2 reduced the time to enter S phase to 36 hours. We then examined the expression of cell cycle-regulatory proteins, p19, p27, cyclins A, D1 and E. Western blots in Figure 4D show that anti-CD3/CD28 stimulation down-regulated p27 protein. The results also showed that stimulation in the presence of IL-2 further up-regulated cyclins A, D1 and E, but not p19. The chemical (**N′-((4-Oxo-4H-chromen-3-yl)methylene) nicotinohydrazide**) has been shown to block STAT5/STAT5 DNA binding activity and inhibit STAT5 tyrosine phosphorylation [35]. We further checked the Cyclin E protein expression after administration of the STAT5 inhibitor to the anti-CD3/CD28- and IL-2-stimulated CD8^+^ T cells. The results revealed that the amount of Cyclin E protein of stimulated CD8^+^ T cells was diminished upon treatment with STAT5 inhibitor, indicating the up-regulated expression of Cyclin E protein by IL-2 signals is dependent on the phosphorylation of STAT5 (Figure 4E). These data suggest that IL-2 helps CD8^+^ T cell activation by up-regulating cyclins A, D1 and E and down-regulating p27.

### IL-2 Signaling during Priming Helps CD8^+^ T Cells Acquiring Effector Functions

**Figure 5:**
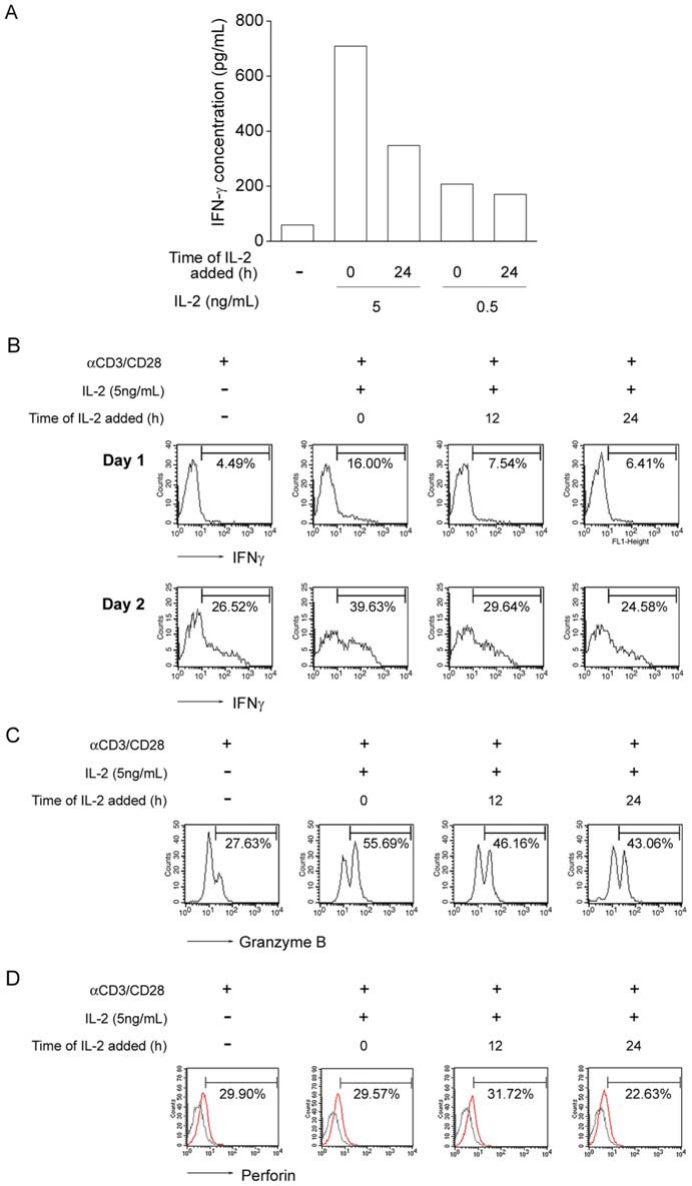
IL-2 signals at priming program CD8^+^ T-cell differentiation. (A) At 48 hours after anti-CD3/CD28 antibodies stimulation, IFNγ level was checked by ELISA. Naïve CD8^+^ T cells were stimulated by anti-CD3/CD28 in the presence or absence of rhIL-2 (5 ng/mL and 0.5 ng/mL) treatment at 0 hour and 24 hours, respectively. (B) At 24 and 48 hours after anti-CD3/CD28 stimulation, production of IFNγ in CD8^+^ T cells was checked by intracellular staining and analyzed by flow cytometry. rhIL-2 (5 ng/mL) was added at 0, 12 and 24 hours after stimulation, respectively. Percentage of IFNγ-producing cells was shown. (C) At 48 hours after anti-CD3/CD28 stimulation, granzyme B production was checked by intracellular staining and analyzed by flow cytometry. Percentage of granzyme B-producing cells was shown. (D) At 24 hours after anti-CD3/CD28 stimulation, perforin production was checked by intracellular staining and analyzed by flow cytometry. Percentage of perforin-producing cells was shown. Similar results were obtained in three independent experiments.

To determine whether IL-2 helps CD8^+^ T cells to acquire effector functions, we examined their IFNγ, granzyme B and perforin productions after priming. Figure 5A shows that CD8^+^ T cells stimulated in the presence of IL-2 produced higher concentrations of IFNγ than those without IL-2 treatment, and IL-2 added at 0 hour of stimulation had greater effect on IFNγ production than that added at 24 hours after stimulation. Moreover, there were a higher percentage of IFNγ-producing cells in the culture with IL-2 added at 0 hour of stimulation (Figure 5B). IL-2 treatment also increased the percentage of granzyme B-producing cells in the CD8^+^ T cell population (Figure 5C). Yet, the level of perforin expression was not increased in cells with IL-2 treatment (Figure 5D). These data together indicate that IL-2 signaling during priming helps CD8^+^ T cells acquire effector functions.

### Generation of Potent CD8^+^ T Cell Anti-Tumor Effector Function Depends upon IL-2 Help

**Figure 6:**
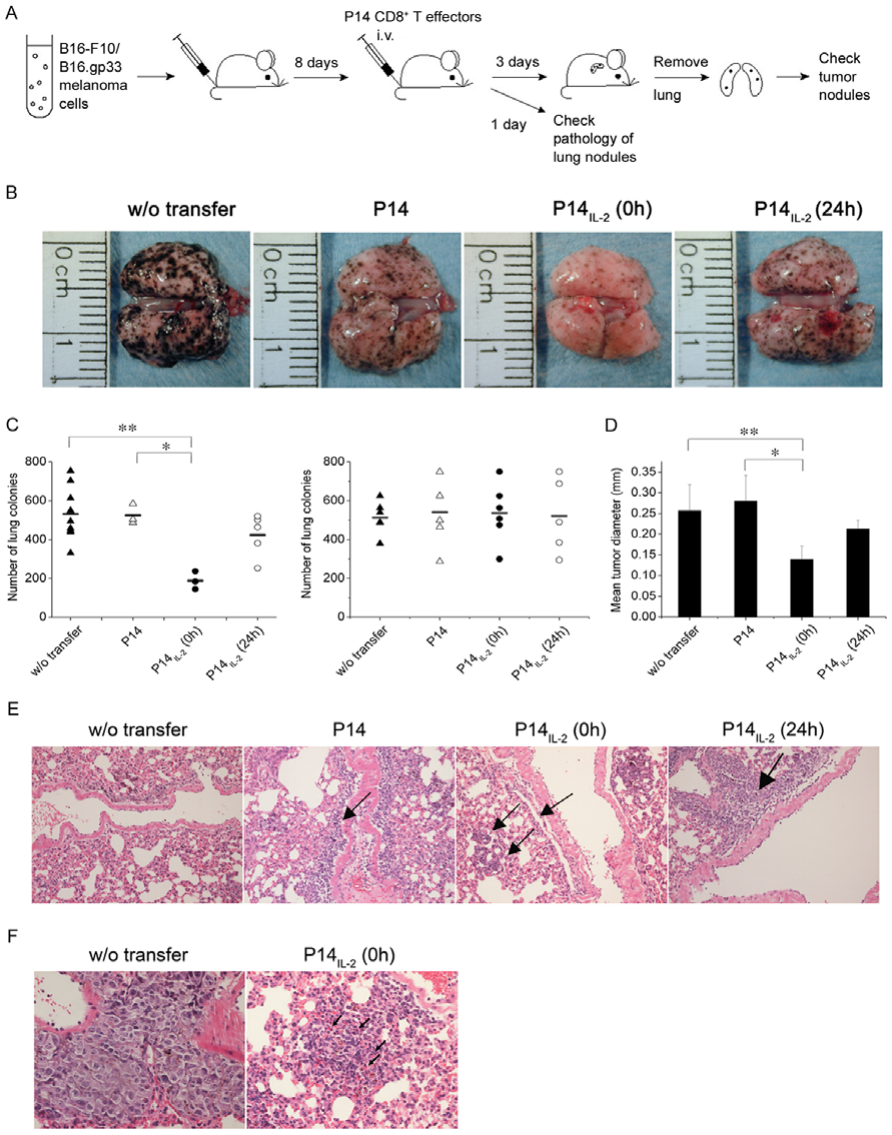
IL-2 signal *in vitro* at CD8^+^ T-cell priming may enhance better tumor-eradicating efficacy *in vivo*. (A) B16-F10 or B16.gp33 melanoma cells were inoculated intravenously into host mice (0.5×10^6^ cells per mouse), followed by injection of P14 effector T cells (1×10^7^ cells per mouse) at 8 days after the tumor injection. The mice were sacrificed one day later for pathology study of lung nodules, or three days later for checking lung tumor nodules. (B) Three days after P14 effector cells transfer, the lungs of two mice in each group was shown. (C) Three days after P14 effector cells transfer, the number of B16.gp33 (left) and B16-F10 (right) melanoma colonies in lung was recorded. (D) Three days after P14 effector cells transfer, the size of B16.gp33 melanoma colonies in lung was recorded. (E and F) One day after P14 effector cells transfer, the lung was fixed in formalin, followed by H& E staining. The pathology of lung nodules was visualized by microscopy at 100× (E) and 400× (F) of magnification. w/o transfer, without cell transfer. P14, transfer with P14 CD8^+^ T effector cells in absence of IL-2 at priming, P14_IL-2_ (0 h) and P14_IL-2_ (24 h) in presence of IL-2 at 0 hour and 24 hours after stimulation, respectively. Similar results were obtained in three independent experiments. *, **, significantly statistical difference by student *t*- test, *p<0.05*. *Arrow in (E), lymphocytes*. *Arrow in (F), apoptotic body*.

To assess whether the IL-2 signal *in vitro* at CD8^+^ T-cell priming may enhance CTL activity *in vivo*, we adoptively transferred activated P14 effector T cells of which the T-cell receptor is specific for MHC class I-restricted GP33 epitope of lymphocytic choriomeningitis virus (LCMV). The P14 CD8^+^ effector cells which were generated by *in vitro* stimulation with the specific peptide, KM9 (KAVTNFATM) in the presence (P14_IL-2_) or absence (P14) of IL-2. The P14_IL-2_ or P14 T effector cells were then adoptively transferred to B16-F10 or B16.gp33 melanoma-bearing mice at 8 days after tumor cell inoculation (Figure 6A). At 11 days after tumor cell inoculation, the lungs were removed and shown in figure 6B. And, the number and the size of melanoma nodules in lungs were recorded. The results showed that adoptive transfer of P14 T effector cells treated with IL-2 at 0 hour after peptide stimulation (P14_IL-2_ (0 h)) caused a significant reduction in the number (Figure 6C, left panel) and the size (Figure 6D) of pulmonary nodules compared to transfer of P14 T cells treated with IL-2 starting at 24 hours (P14_IL-2_ (24 h)) after peptide stimulation and that without IL-2 treatment (P14). While, transfer of the effector cells did not show tumor elimination in B16.F10-bearing mice (Figure 6C, right panel).

**Figure 7:**
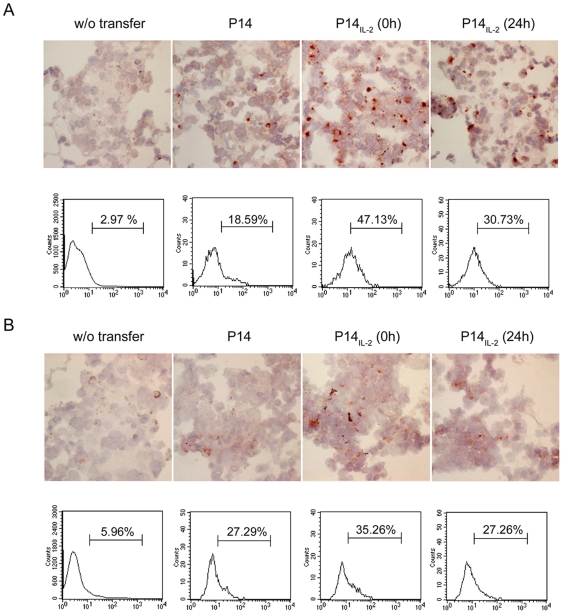
IL-2 signal at priming drove better anti-tumor CTL function *in vivo*. (A) Expression of granzyme B in tumor-infiltrating lymphocytes. *Upper panel*. At 4 hours after effector cells transfer, the lung tissue was subjected to immunohistochemical staining by FITC anti-granzyme B antibody (1 µg/mL), followed by anti-Fluorescein peroxidase. After wash, the tissue was subjected to NOVA-RED and Hematoxylin staining. *Lower panel*. At 4 hours after transfer of CFSE-labeled effector cells, the lymphocytes in the lung were isolated and subjected to intracellular staining by PE anti-granzyme B antibody, and analysis by flow cytometry. (B) Expression of IFNγ in tumor-infiltrating lymphocytes. *Upper panel*. At 4 hours after effector cells transfer, the IFNγ production in the lung was checked by immunohistochemical staining by anti-IFNγ antibody (5 µg/mL), followed by biotin goat anti-rat Ig (1 µg/mL) and streptavidin-peroxidase (1∶1000). After wash, the tissue was subjected to NOVA-RED and Hematoxylin staining. *Lower panel*. As (A), the production of IFNγ was also checked in the CFSE-labeled effector cells using intracellular staining by PE anti-IFNγ antibody, and analysis by flow cytometry. Similar results were obtained in three independent experiments.

H & E stain revealed that there were lymphocytes migrating out of the circulation to tumor sites in mice receiving effector T cells at one day after adoptive transfer (Figure 6E). Of note, more lymphocytes infiltrated into the tumor sites in the mice receiving P14_IL-2_ (0 h) T cells than that in mice receiving P14 (Figure 6F). Apoptotic bodies were also found in the tumor (Figure 6F, arrow). To test the hypothesis that the more potent tumor eradication in mice receiving P14_IL-2_ (0 h) effector cells is caused by greater cytolytic function of the cells transferred, we checked the production of granzyme B and IFNγ of the tumor-infiltrating lymphocytes of effector cells. At 4 hours after effector cells transferred, the lung was resected and stained with anti-Granzyme B and anti-IFNγ antibodies by immunohistochemical staining (Figure 7A and 7B, upper panel). The results showed that there were more Granzyme B- and IFNγ- producing cells in the tumor in mice receiving P14_IL-2_ (0 h) than other groups. We also labeled the effector cells with CFSE before transfer and isolated the lymphocytes in the lung. The expression of granzyme B and IFNγ of the CFSE-labeled cells were then checked by intracellular staining and analyzed by flow cytometry (Figure 7A and 7B, lower panel). We found that greater percentage of tumor-infiltrating P14_IL-2_ (0 h) effector T cells produced granzyme B and IFNγ than P14 and P14_IL-2_ (24 h) effector T cells (granzyme B: 47.13% vs. 18.59% vs. 30.73%, IFNγ: 35.26% vs. 27.29% vs. 27.26%). These results demonstrate that P14 effector T cells generated in the presence of IL-2 from 0 h on attained better effector function and were highly effective in reducing tumor burden *in vivo*. Exposing P14 T cells to IL-2 at later time point (24 hours after stimulation) during peptide stimulation generated P14 effector T cells with only moderate therapeutic efficacy *in vivo*. It clearly indicates that IL-2 exposure and the temporal function of IL-2 during TCR stimulation control the potency of the CD8^+^ effector T cells generated.

## Discussion

CD8^+^ T cells are key effector cells to fight against tumor and viral infections. It is crucial that CD8^+^ T cells proceed to undergo expansion and acquire full effector functions upon activation through specific T cell receptor and costimulatory molecules. Previous studies have shown that CD4^+^ T-cell help is required for the generation of functional CD8^+^ T-cell responses [13], [14], [15], [18], [23], [24], [25]. However, it is not clear at what point during CD8^+^ T cell priming that CD4^+^ T-cell help is needed. This paper describes our findings on the timing and how CD4^+^ T cells provide help to CD8^+^ T cells that result in functionally activated effector cells.

We showed that by secreting IL-2, activated CD4^+^ T cells provide help to CD8^+^ T cells. It was the signals transduced through IL-2Rβγ within the first 0–2.5 hours that IL-2 helps CD8^+^ T cell activation. The activation of IL-2 signaling advances the restriction point of cell cycle, and thereby expedites the entry of stimulated CD8^+^ T cells into the S phase. Besides promoting CD8^+^ T cells proliferation, IL-2 stimulation also augments IFNγ and granzyme B production. Importantly, effector CD8^+^ T cells generated *in vitro* in the presence of IL-2 at the priming stage proved to be fully functional in anti-tumor activity *in vivo*. As a result, CD4^+^ T-cell help represents a ‘checkpoint’ in triggering a CD8^+^ T-cell response and insures a productive CD8^+^ T-cell response occurs only under appropriate circumstances. Our work demonstrated the temporal function of CD4^+^ T cell-derived IL-2 for governing the activation and differentiation of CD8^+^ T cells.

IL-2 holds several functions for T cells. It has been thought for decades that IL-2 not only serves as a growth factor for T cells following activation [18], [19], but also prevents T cells from anergy [36], [37]. One recent study showed that IL-2 signals to pathogen-specific CD8^+^ T cells during primary infection are crucial for the generation of robust secondary expansion of CD8^+^ memory T cells [38]. However, less is known how it acts to shape the CD8^+^ T-cell response in priming phase. One recent report has shown that naive CD8^+^ T cells preferentially accumulated in lymph nodes and attracted to the sites of antigen-specific dendritic cell-CD4^+^ T cell interaction. Thus, the naïve CD8^+^ T cells were kept in close proximity to activated CD4^+^ T cells. This allows the immune system to efficiently activate the naïve CD8^+^ T cells [39]. Our study and other reports showed that CD4^+^ T cells secret significant amount of IL-2 soon immediately after activation (Table 1) [40], [41]. It is plausible for naïve CD8^+^ T cells to expose to a high concentration of IL-2 locally when they are encountering the specific antigen. It is noteworthy that naïve CD8^+^ T cells only bear IL-2Rβγ, the intermediate affinity receptor for IL-2 [33] and the α-chain of IL-2 receptor, CD25, is not expressed on naïve CD8^+^ T cells and is significantly up-regulated until 6 hours after stimulation (Figure 3E). It is plausible that IL-2 at priming delivers a functional signal through IL-2Rβγ to up-regulate CD25 expression, which further ensures the antigen-stimulated CD8^+^ T-cell proliferation.

We also showed that IL-2 alone was not able to initiate CD8^+^ T-cell proliferation, and to elicit a full activation of CD8^+^ T cells occurs only when antigen is present. Activation of the TCR renders the cells “competent” to receive the cell-cycle progression signals that are provided by IL-2. Once IL-2 signals at CD8^+^ T-cells priming is transduced, the cell cycle-governing proteins such as cyclin A, cyclin D1, and cyclin E are up-regulated. In this respect, antigen-stimulated cells which undergo G0 to G1 transition can thus progress through G1 to S phase without delay when IL-2 is also provided. However, when IL-2 is provided at later time, the cells may require more time to enter S phase. Or, the cells may be arrested until later production of autocrine IL-2 to precede the process. Furthermore, IL-2 at priming also induces up-regulation of CD25 expression, which further ensures the signal transduction of IL-2 in the antigen-stimulated CD8^+^ T-cells. Not only the impact on cell cycle, IL-2 provided at early priming also enhanced CD8^+^ T cells effector function. The production of IFNγ from activated CD8^+^ T cells increased at 48 hours after stimulation whenever IL-2 added at early or later time. But the concentration of IFNγ from CD8^+^ T cells added IL-2 at 24 hours after stimulation was not as high as that in IL-2 added at 0 hour (Figure 5A). And, IL-2 added at early priming rendered more IFNγ–producing cells in the population. However, the percentage of IFNγ–producing cells did not increase significantly at 48 hours later when IL-2 provided at 12 or 24 hours after stimulation (Figure 5B). These suggest that IL-2 signals at early priming may expedite the CD8^+^ T cells to attain full differentiation. Accordingly, the antigen-stimulated CD8^+^ T-cells with later IL-2 administration may need longer time to produce higher amount of IFNγ than that IL-2 added at early priming (Figure 5A and B). The similar pattern was also shown in Granzyme B-producing activated CD8^+^ T cells (Figure 5C) but not in perforin-producing cells (Figure 5D). Taken together, when encountering their antigen, the IL-2 signals at priming not only ensure the antigen-stimulated CD8^+^ T cells to progress the cell cycle, but may also advances the response faster. Therefore, the present study suggests that the temporal function of IL-2 signal is critical for the antigen-stimulated CD8+ T cells to attain a full activation.

T-cell adoptive immunotherapy is a treatment strategy for cancer. Despite the fact that a coordinated effort by modifications in culture technique and administration of nonmyeloablative chemotherapy has markedly improved of the clinical response in melanoma patients, it remains poorly understood why the tumor regression tend to be partial. To achieve the best therapeutic efficacy, it is crucial to adoptive transfer the CTL with good effector function. Our data showed that IL-2 signal at priming stage *in vitro* drove development of CD8^+^ effector cells which produced greater quantities of IFNγ and Granzyme B when encountered the tumor *in vivo*. The more tumor-infiltrating lymphocytes were also observed in the group of IL-2 adding at priming. The results reported here demonstrate that IL-2 signal in the early priming stage not only drove the CD8^+^ T-cell differentiation but also aid to maintain effector function *in vivo*.

On the basis of the findings of this study, the requirement of CD4^+^ T-cell help for regulation of CD8^+^ T-cell immune responses should be reconsidered more precisely. It is speculated that following activation, CD4^+^ T cells secrete IL-2 to directly help CD8^+^ T-cell priming. As a consequence, the antigen-stimulated CD8^+^ T cells attain full activation and differentiation. It reflects that CD4^+^ T-cell help promotes a net gain of stimulation for those neighboring CD8^+^ T cells. Interestingly, IL-2 signals exclusively to antigen-encountered CD8^+^ T cells also confine the immune response in local inflammatory sites. As a result, undesired systemic immune responses can be limited. Elucidation of the mechanism will aid to better understand the development of CD8^+^ T-cell activation and differentiation and to optimize vaccine strategies for inducing protective immunity.

## Materials and Methods

### Mice

All procedures were performed following the guideline of the Use of Laboratory Animals published by National Taiwan University and approved by Institutional Animal Care and Use Committee of College of Medicine and College of Public Health of National Taiwan University. C57BL/6 mice were obtained from the animal center at National Taiwan University Hospital and maintained in pathogen-free conditions. The 2C and P14 TCR-transgenic mice were kindly obtained from Dr. John T. Kung (Academia Sinica, Taipei, Taiwan). 2C transgenic mice carries functional rearranged TCR α-(one copy) and β-(eight copies) chain transgenes from a cytotoxic T-cell clone 2C which is specific for L^d^ MHC class I antigen. P14 transgenic mice carries rearranged TCR transgenes specific for the gp33 epitope (amino acid 33–41 of glycoprotein) of lymphocytic choriomeningitis virus (LCMV). All animals were maintained in the specific pathogen-free facility and were used at age of 4–6 weeks.

### Cell Preparation and Culture Conditions

#### Cells

Naïve CD62L^hi^CD44^lo^CD8^+^ T cells used for experiments were obtained from spleens of either C57BL/6 mice or 2C TCR-transgenic mice specific for QL9 peptide.

#### Isolation of naïve CD8^+^ T cells

Single-cell splenocytes suspensions were twice depleted of Ig^+^ B cells by adherence to culture plates coated with anti-Igμ mAb and anti-Igκ mAb for 60 min at room temperature. Nonadherent cells were collected and CD8^+^ T cells with purity above 95% were enriched by incubated in anti-CD8 mAb (clone 3.155)-coated culture plates. CD62L^hi^ cells were then positively isolated by magnetic cell sorting. The resultant CD8^+^CD62L^hi^CD44^low^ T cells with purity above 93% were used for further experiments.

#### Isolation of naïve CD8^−^ splenocytes

Single-cell splenocytes suspensions were twice depleted of CD8^+^ T cells by adherence to culture plates collated with anti-CD8 mAb (clone 3.155) for 60 min at room temperature. The nonadherent cells were collected and less than 0.5% of CD8^+^ T cells were detected in the cell populations.

#### Isolation of CD4^+^ T cells

Single-cell splenocytes suspensions were twice depleted of Ig^+^ B cells by adherence to culture plates coated with anti-Igμ mAb and anti-Igκ mAb for 60 min at room temperature. Nonadherent cells were collected and CD4^+^ T cells with purity above 95% were enriched by incubated in anti-CD4 mAb (clone GK1.5)-coated culture plates.

#### Primary CD8^+^ T cell stimulation

All naïve CD8^+^ T cells activation cultures were set up in DMEM containing 10% FBS, 50 mM HEPES and 5×10^−5^ M 2-ME in standard 24- or 96-well microculture plates (Corning Inc.). To activate T cells, the naïve CD8^+^ T cells were incubated in 96- or 24-well plate in the presence of 1 µg/mL rabbit anti-hamster (R anti-H) plus anti-CD3 mAb (clone 145-2C11) and anti-CD28 mAb (clone PV1) for the indicated time. For activating 2C transgenic CD8^+^ T cells, the cells were incubated with mitomycin C-treated (inhibition of DNA synthesis) [27], [28], [29] LPS-stimulated B blasts [42], [43] from B10.A mice and QL9 peptide (QLSPFPFDL, Proimmune Inc., Oxford, United Kingdom) in titrated concentrations. Recombinant murine IL-2 and recombinant human IL-2 were administrated at a final concentration of 5 ng/mL (equivalent to 100 IU/mL). Cell numbers were determined by trypan blue exclusion. Cells were grown at 37°C and 5% CO_2_ in humidified air.

### Collection of Culture Supernatants

Total splenocytes, CD8^−^ splenocytes and CD4^+^ T cells were stimulated by immobilized anti-CD3 plus soluble R anti-H and anti-CD28 antibodies for indicated time, 0, 2, 4 and 24 hours. The culture supernatants were collected for further experiments.

### Antibodies and Reagents

DMEM and penicillin and streptomycin from GIBCO Inc.(Grand Island, NY, USA), and Fetal bovine serum (FBS) from HyClone Lab Inc.(Logan, UT, USA) were purchased. Hamster anti-mouse CD3 monoclonal antibody (clone 145-2C11), hamster anti-mouse CD28 monoclonal antibody (clone PV1), rat anti-mouse CD4 monoclonal antibody (clone GK1.5) were prepared in our laboratory. Anti-mouse Igμ (clone Bet.2) and anti-mouse Igκ (clone 187.1) and rat anti-mouse CD8 monoclonal antibodies (clone 3.155) were gifts from Dr. John T. Kung (Academia Sinica, Taipei, Taiwan). Anti-mouse IL-2 antibody (clone S4B6), isotype control rat IgG_2a_, anti-mouse IFNγ (clone XMG1.2), FITC anti-mouse granzyme B (clone 16G6), PE anti-mouse perforin (clone eBioOMAK-D, eBioscience, San Diego, CA, USA), FITC anti-mouse CD3, PE anti-mouse CD4, PE-Cy5 anti-mouse CD8, FITC anti-mouse CD25, PE anti-mouse CD69, FITC anti-CD122, FITC Annexin V, mouse anti–p27 antibodies and BrdU kit (BD Pharmingen, San Diego, CA, USA), rat anti-CD122 antibody (TM-β1) and isotype control rat IgG_2b_ (eBioscience, San Diego, CA, USA), rabbit polyclonal anti-HIg antibody (R anti-H, Jackson ImmunoResearch Laboratories, West Grove, PA, USA), mouse anti-cyclin E antibody (Santa Cruz Biotechnology, Santa Cruz, CA, USA), mouse anti-actin (Chemicon, Temecula, CA, USA) were used in the experiments. MTT (3-[4,5-dimethylthiazol-2-yl]-2,5-diphenyltetrazolium bromide), mitomycin C and propidium iodide (Sigma, St Louis, MO, USA) were purchased. Anti-phospho-stat5 and anti-stat5 antibodies were purchased from Cell Signaling Technology, Inc. (Danvers, MA, USA). Goat polyclonal anti-rabbit and anti–mouse IgG–horseradish peroxidase were purchased from Amershan Corp (Little Chalfont, Buckinghamshire, United Kingdom).

### Cell Proliferation Assays

#### MTT assay (colorimetric assay)

MTT is especially useful for assaying the quantification of viable cells, because MTT is cleaved to form a formazan dye (purple color) only by metabolic active cells [44], [45], [46]. Following stimulation for 96 hours, the cells were incubated with MTT (1 mg/mL) for another 4 hours. The formazan was solubilized by dimethyl sulfoxide and colorimetric absorbance was quantified by measuring the optical density (OD) at 570 nm by a spectrophotometer (Tecan Group Ltd., Männedorf, Switzerland).

#### CFSE (carboxyfluorescein succinimidyl ester) labeling

Before stimulation, the naïve CD8^+^ T cells were resuspended at a concentration of 10×10^6^ cells/mL in 0.1% bovine serum albumin (BSA) in PBS and CFSE (Molecular Probes, Eugene, OR, USA) was added to a final concentration of 5 µM. After 10 min of incubation at 37°C, labeling was quenched with ice-cold DMEM. Cells were washed and resuspended in DMEM for further culture. At the indicated time following stimulation, the cells were analyzed by flow cytometry.

#### BrdU incorporation

BrdU (10 µM) was added to cell cultures during the final 60 min. Cells were harvested and fixed and resuspended in DNase I solution as described [47]. To stain BrdU, FITC anti-BrdU antibody (1 µg/mL) was used for 30 min at 4°C and washed by FACS buffer (2% FBS in 1X PBS). The cells were analyzed by flow cytometry.

### Expression of Cyclins and STAT 5 Inhibition

The naïve CD8^+^ T cells were incubated with anti-CD3/CD28 antibodies in the presence of absence of rhIL-2 (5 ng/mL). At the indicated time, 18 and 24 hours after stimulation, the cells were lysed with modified RIPA solution (50 mM tris-HCl [pH 7.4], 150 mM NaCl, 1 mM EDTA, 1% NP-40, 0.25% Na-deoxycholate, 1 mM PMSF, 1 mM orthovanadate and 0.1% SDS) with a protease inhibitor cocktail (Roche, Mannheim, Germany) [48] and 20 µg of the protein was subjected to SDS PAGE, followed by immunoblotting. To inhibit stat5 phosphorylation, the cells were pretreated with the stat5 inhibitor (N′-((4-Oxo-4H-chromen-3-yl)methylene) nicotinohydrazide, Calbiochem Inc.) [35] in titrated concentrations for 15 minutes, followed by stimulation with anti-CD3/CD28 antibodies and IL-2.

### Measurement of IL-2, IFNγ, Granzyme B and Perforin

The amounts of murine IL-2 or IFNγ in 100-µL culture supernatants were determined by enzyme-linked immunosorbent assay (ELISA) according to the manufacturer's instructions (BD Pharmingen, San Diego, CA, USA). The detection limit of the assay was 0.04 ng/ml. The production of murine IL-2, IFNγ, granzyme B and perforin were also checked by intracellular staining. The antibody specific for murine IL-2 was not reactive toward the rhIL-2 in the culture.

### Adoptive Immunotherapy

#### B16.gp33 melanoma cells

B16.gp33 melanoma cells derived from B16 melanoma cells genetically modified to express gene encoding amino acid 33–41 of glycoprotein from LCMV (gp33) under the control of actin promoter were kindly provided by Dr. Hanspeter Pircher [49]. B16.gp33 melanoma cells were inoculated intravenously into host mice (0.5×10^6^ cells per mouse). Tumor colonies with characteristic dark pigmentation formed in the lungs after intravenous inoculation. Tumor diameter was also measured as an index of tumor growth.

#### Generation of P14 CTL

P14 TCR is specific for amino acid 33–41 of the glycoprotein from LCMV (gp 33 epitope of LCMV) in the context of H-2D^b^. P14 CTL were generated by activation of P14 CD8^+^ T cells from the spleens as previously described [39]. P14 CD8^+^ T cells (1×10^5^ cells/mL) were activated by KM-9 antigenic peptide (KAVTNFATM; AnaSpec, San Jose, CA) presented by LPS-activated syngeneic B cell blasts (mitomycin C treated; 5×10^5^ cells/mL), with or without rhIL-2 (5 ng/ml) administration at 0 or 24 hours after stimulation. At the end of the 3-day activation culture, the activated cells were washed and cultured in the presence of rhIL-2 (5 ng/mL) for 3–4 more days.

#### P14 CTL adoptive immunotherapy

The P14 effectors were injected via the tail vein in 0.15 mL of 1 X PBS into the mice that had previously been inoculated with B16.gp33 melanoma cells for 8 days. Control mice for the adoptive transfer received 1 X PBS or B16-F10 cells which did not express gp33 epitope of LCMV.

### Detection of Granzyme B and IFNγ in Tumor-Infiltrating Lymphocytes

At 4 hours after transfer of CFSE-labeled P14 and P14_IL-2_ effector cells, the lung tissue was subjected to immunohistochemical staining by FITC anti-granzyme B antibody (1 µg/mL), followed by anti-Fluorescein peroxidase. For checking IFNγ production in the lung by immunohistochemical staining, the lung tissue was stained with anti-IFNγ antibody (5 µg/mL), followed by biotin goat anti-rat Ig (1 µg/mL) and streptavidin-peroxidase (1∶1000). After wash, the tissue was subjected to NOVA-RED and Hematoxylin staining. In addition, the lymphocytes in the lung were isolated and subjected to intracellular staining by PE anti-granzyme B or PE anti-IFNγ antibody, and analysis by flow cytometry. For isolation of lymphocytes in the lung, lung tissues were ground with fine metal strainer, followed by digestion with HBSS containing 5% FBS, 125 U/mL collagenase I (GIBCO Inc., Grand Island, NY, USA), 60 U/mL DNase I and 60 U/mL hyaluronidase (Sigma, St Louis, MO, USA) for 60 min at 37°C. After wash with HBSS for 3 times and RBC lysis, the cells were harvested for further intracellular staining.

### Statistical Analysis

Experiments were performed in triplicates for at least three times. Data are presented as mean values±S.D. Statistical analysis of the data was carried out using *Student t* test.
